# Diagnostic Value of microRNA-375 as Future Biomarker for Prostate Cancer Detection: A Meta-Analysis

**DOI:** 10.3390/medicina58040529

**Published:** 2022-04-10

**Authors:** Diana Nitusca, Anca Marcu, Edward Seclaman, Razvan Bardan, Ioan Ovidiu Sirbu, Ovidiu Balacescu, Adina Ioana Bucur, Sorin Ursoniu, Catalin Marian

**Affiliations:** 1 Department of Biochemistry and Pharmacology, Victor Babes University of Medicine and Pharmacy, Pta Eftimie Murgu Nr. 2, 300041 Timisoara, Romania; nitusca.diana@umft.ro (D.N.); marcu.anca@umft.ro (A.M.); eseclaman@umft.ro (E.S.); ovidiu.sirbu@umft.ro (I.O.S.); cmarian@umft.ro (C.M.); 2 Center for Complex Networks Science, Victor Babes University of Medicine and Pharmacy, Pta Eftimie Murgu Nr. 2, 300041 Timisoara, Romania; 3 Department of Urology, Victor Babes University of Medicine and Pharmacy, Pta Eftimie Murgu Nr. 2, 300041 Timisoara, Romania; razvan.bardan@umft.ro; 4 Urology Clinic, County Emergency Clinical Hospital “Pius Brînzeu”, Bd. L. Rebreanu Nr. 156, 300723 Timisoara, Romania; 5 Department of Functional Genomics, Proteomics and Experimental Pathology, The Oncology Institute “Prof. Dr. Ion Chiricuta”, Str. Republicii Nr. 34-36, 400015 Cluj-Napoca, Romania; ovidiubalacescu@iocn.ro; 6 Department of Functional Sciences, Discipline of Public Health, Victor Babes University of Medicine and Pharmacy, Pta Eftimie Murgu Nr. 2, 300041 Timisoara, Romania; bucur.adina@umft.ro; 7 Center for Translational Research and Systems Medicine, Victor Babes University of Medicine and Pharmacy, Pta Eftimie Murgu Nr. 2, 300041 Timisoara, Romania

**Keywords:** prostate cancer, microRNAs, miR-375, diagnostic, biomarker

## Abstract

*Background and Objectives:* Responding to the need for additional biomarkers for the diagnosis of prostate cancer (PCa), mounting studies show that microRNAs (miRNAs/miRs) possess great potential as future promising diagnostic tools. However, the usefulness of these miRNAs is still highly debated, as the degree of inconsistency between study designs and results is still elevated. Herein, we present a meta-analysis evaluating the diagnostic value and accuracy of circulating miR-375, as it is one of the most studied types of miRs in PCa. *Materials and Methods:* The diagnostic accuracy of miR-375 was evaluated using the QUADAS-2 tool, analyzing different statistical parameters. The seven studies (from six articles) that matched our selection included 422 PCa patients and 212 controls (70 healthy volunteers + 142 with benign prostate diseases). *Results and Conclusion:* We obtained a *p*-value of 0.76 for sensitivity, 0.83 for specificity, 16 for DOR, 4.6 for LR+, 0.29 for LR−, and 0.87 for AUC (95% CI 0.83–0.89). Our results confirm that miRNA-375 has high diagnostic potential for PCa, suggesting its usefulness as a powerful biomarker. More comprehensive studies are warranted to better assess its true value as a diagnostic biomarker for this urologic disease.

## 1. Introduction

One of the most frequent causes of cancer in males in the U.S. alone is represented by prostate cancer (PCa), accounting for 191,930 estimated new cases in 2020, with 33,330 new deaths [1]. Over the course of time, there is an increasing incidence of this urologic malignancy, which appears to be related at least partly because of the ubiquitous use of prostate-specific antigen (PSA) screening, which has shown to be problematic in terms of specificity, ultimately leading to overdiagnosis and unnecessary biopsies [2,3]. Substantial research effort is being undertaken to better understand the mechanisms of PCa, with tremendous progress being achieved in the last decade [4,5]. However, there is still a pressing need for the discovery of novel, optimized, and more accurate diagnostic biomarkers that could represent useful candidates for clinical application.

MicroRNAs (miRNAs/miRs) are a class of short-length, non-coding RNA molecules, highly involved in physiology. They regulate gene expression after transcription by complementarily binding to target specific sequences of corresponding messenger RNAs (in the 3′UTR region), thereby regulating gene expression in two ways: mRNA degradation or protein translation inhibition [6,7]. Moreover, the expression of numerous miRNAs appears to be dysregulated in PCa regardless of stage [8]. Therefore, given their differential expression and their stability in biological fluids, miRNAs have been proposed as suitable, minimally invasive cancer biomarkers [9,10,11].

MiR-375 (located on chromosome 2q35) was first discovered in pancreatic islets as a β-cell regulator for function and development and in insulin secretion by targeting MTPN and PDK-1 genes. Last-decade PCa research describes miR-375 as oncogenic and associated with metastatic castration-resistant PCa as well as with biochemical recurrence (defined as a rise in serum PSA levels following radical prostatectomy and/or radiation therapy). Gene ontology analysis revealed QKI, EHMT1, and JAK2 as potential target genes for miR-375, which are found to be mainly involved in ubiquitin-mediated proteolysis and protein binding, suggesting therefore a potential role in the aggressive type of PCa [12]. Furthermore, although the main function of miR-375 has not been characterized, a ZEB1-miR-375-YAP1 signaling pathway has been identified to regulate epithelial plasticity in PCa by Selth et al., who also discovered that miR-375 was highly and positively associated with tumor cells in the metastatic-stage PCa, thus bringing insight into tumor cell invasion mechanisms of PCa in relationship with miR-375 expression [13].

In addition, miR-375 is categorized among the most frequently studied types of miRs in PCa, with a potentially promising future as a candidate biomarker for PCa detection [14,15]. However, although mounting evidence links miR-375 not only with PCa carcinogenesis, progression, and pathophysiology but also with its relevance as a candidate biomarker, the true diagnostic value of this miR has not been assessed in PCa alone, with studies investigating its diagnostic significance only in the context of multiple human cancers or within a panel encompassing various miR species. In addition, the study of miR species as non-invasive circulating biomarkers for cancer detection from liquid biopsy (i.e., plasma, serum, urine) further narrows down the sample size of a meta-analysis, as the great majority of studies are analyzing miR expression using tissue samples as biological specimens (which require invasive biopsy procedures). Nonetheless, inconsistent results have been obtained across studies due to differences between study designs [9,16,17,18,19,20,21,22,23,24]. It appears that the degree of heterogeneity between studies remains high, making the design of a meta-analysis a difficult task. For example, the specificity for miR-375 varies considerably among studies: 1 and 0.39 for Haldrup et al. and Stuopelyte et al., respectively [22,23], and the sensitivity for miR-375 varies between 0.23 and 1 [17,22]. Such differences in specificity and sensitivity values could arise due to the use of divergent sample populations (cohort sizes; different controls used whether healthy and/or with benign disease; varying PCa stages), diverse technical and methodological approaches regarding miR-375 extraction protocols, PCR quantification and endogenous controls used for data normalization, as well as the choice of downstream statistical analysis software and interpretation.

Therefore, considering the inconsistencies that we found among studies, we conducted herein the first meta-analysis to accurately assess the diagnostic value of miR-375 only in PCa.

## 2. Materials and Methods

We conducted our meta-analysis based on the PRISMA 2020 statement guidelines (Table S1) [25].

### 2.1. Electronic Search Procedure

All reports included in our study were found by two independent examiners via *PubMed, Embase*, and *Web of Knowledge* databases research (through March 2022) using the following keywords: (“microRNA 375” or “miRNA-375” or “miR-375”) and (“circulating” or “blood” or “serum” or “plasma” or “urine”) and (“prostate”) and (“diagnosis” or “sensitivity” or “specificity” or “ROC curve”). The references of the articles of interest were analyzed to identify other relevant reports. The aforementioned medical subject headings (MeSH) were joined together using “AND” and “OR” functions (all fields).

### 2.2. Eligibility Criteria

We selected studies that included patients with diagnosed PCa and controls that were either healthy or with benign prostatic hyperplasia (BPH). The index tests for miR-375 from the selected biological samples (serum, plasma, and urine) were performed by quantitative real-time PCR (q-PCR) by firstly reverse-transcribing miR-375 sequence into cDNA, followed by amplification of the reverse-transcribed product using sequence-specific forward and reverse primers and fluorescent probes. Patients were diagnosed with PCa based on histopathological confirmation (reference standard). The diagnosis techniques were mainly done by transrectal ultrasound-guided biopsy (TRUS-biopsy), and Gleason scores were applied based on the International Society of Urological Pathology (ISUP) 2005 recommendations. However, most studies did not offer detailed information regarding the clinical guidelines applied to the histopathological confirmation.

Research articles’ inclusion criteria were the following: (1) reports evaluating the diagnostic performance of miR-375 in circulating samples; (2) inclusion of numerical data on sensitivity, specificity, AUC values, and 2 × 2 contingency tables that could be calculated or extracted; (3) PCa diagnosis was performed via histopathological testing, and the controls were either healthy or with benign prostatic diseases (case-control design); and (4) were published in English.

Research articles’ exclusion criteria were the following: (1) non-original papers (letters, conference abstracts, reviews); (2) studies with insufficient data for our interests; (3) duplicate studies already included; and (4) not written in English language.

With respect to our research inclusion criteria, we discovered that the most common PCa stages present in the included studies were the pathological/clinical stages 1 and 2 (72.9%), usually with no metastases, and therefore, we consider that our downstream statistical results for miR-375 could be included in the screening diagnostic pathway.

### 2.3. Data Extraction

The following information was retrieved or calculated from all reports included in our meta-analysis by the two reviewers (D.N. and A.M.): first author (last name); year of publication; specimen collected; cases (PCa patients); controls (healthy/BPH); miRNA expression profiling method; endogenous control for normalization; relative miR-375 expression in PCa group compared with controls; sensitivity and specificity values; true positives (TPs), false positives (FPs), false negatives (FNs), and true negatives (TNs), all calculated; and AUC with corresponding 95% CI.

### 2.4. Quality Assessment

QUADAS-2 was operated by two examiners in an independent fashion to reliably assess the quality of each included report [17,22,23,24,26,27]. This instrument includes four key domains (selection of patients, index test, reference standard, and flow and timing), all of which are assessed for risk of bias. For the applicability concerns testing, the first three areas were also used. A low risk of bias was taken into consideration if all the questions were answered with “yes”. The risk of bias occurred when one question was answered with “no”. The “?” (unclear) field was applied only for insufficient or poorly reported data to allow for proper judgment. The applicability concerns areas did not encompass these signaling questions but were categorized as “high”, “low”, or “unclear”.

The results of quality assessment were subsequently used to assess the general quality of the studies that were included in our meta-analysis and to examine for possible sources of heterogeneity [28].

### 2.5. Statistical Analysis

All statistical analyses were performed using Stata statistical software MP 15.1 (StataCorp LP, College Station, TX, USA) using the *metandi* and *midas* commands. Statistical significance was considered for a *p*-value lower than 0.05. Heterogeneity between studies was assessed using I^2^ index and χ^2^ test. Heterogeneity was taken into consideration for I^2^ percentages of over 50% (*p* < 0.05). Sensitivity and specificity values were taken out from each individual report to assess the overall accuracy of the miR-375 assays. In addition, our independent examiners calculated the values for the true positives (TPs), true negatives (TNs), false positives (FPs), and false negatives (FNs) in each included report.

Furthermore, we used a random-effects model to calculate pooled positive and negative likelihood ratios (designated as LR + and LR−, respectively) and their corresponding 95% confidence intervals (CIs) [29,30]. LR+ represents the probability of a positive result in a patient with PCa (correctly assigned), while LR− represents the probability of a positive outcome in a disease-free subject (either a healthy volunteer or a patient with benign prostatic disease).

The AUC value was obtained based on the construction of a pooled summary receiver operating characteristics (SROC) curve, which was useful in assessing the true diagnostic performance for miR-375. The SROC plot also contained an overall sensitivity and (1-specificity) estimate across reports together with their 95% CIs and a 95% prediction interval that accounts for heterogeneity.

Forest plots for the sensitivity and specificity values (with 95% CIs) were generated for each individual study [31,32]. For the sensitivity analysis, we performed a quantile plot of the goodness-of-fit based on residuals; a chi-square probability plot of squared Mahalanobis distances to evaluate the bivariate normality supposition; a spike plot to check for influential observations in particular using Cook’s distance; and a scatterplot to verify and remove outliers using random effects (based on residuals—level 2).

Meta-regression analyses were performed to determine the extent of heterogeneity between reports in terms of sample specimens. In addition, in order to investigate the likelihood of publication bias events, we generated a Deeks’ funnel plot, as described [33].

Lastly, Fagan’s nomogram was used to assess the clinical relevance and patient utility of the diagnostic test and to evaluate for post-test probabilities [34].

## 3. Results

### 3.1. Data Selection and Characteristics of Studies

Following database electronic search, a total number of 212 reports were found—out of which, 117 were duplicate studies and were subsequently excluded. The remaining 95 articles were screened for titles and abstracts, and 68 were excluded for being either unrelated to the topic or lacking data for our interests. Next, 27 full-text studies were assessed, out of which 21 did not meet our inclusion criteria: 2 were reviews, and 19 did not offer sufficient statistical data. Finally, six articles, comprising seven different studies, were included in our meta-analysis [17,22,23,24,26,27].

**Figure 1:**
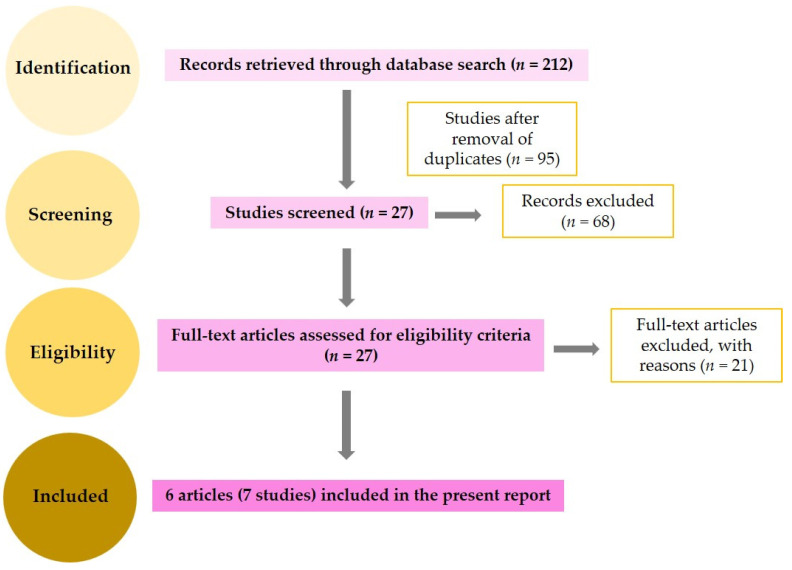
Study selection process (flow-diagram).

We summarize our study selection in the flow diagram of Figure 1. The basic characteristics of the included reports are shown in Table 1. From the six total articles that we included in our meta-analysis, we identified a total of 422 patients with diagnosed PCa and 212 controls (70 healthy subjects and 142 patients with BPH). Among all studies included, three used serum, two used plasma, and two used urine as sample specimens. Studies were published between 2008 and 2020.

### 3.2. Quality Assessment

**Figure 2:**
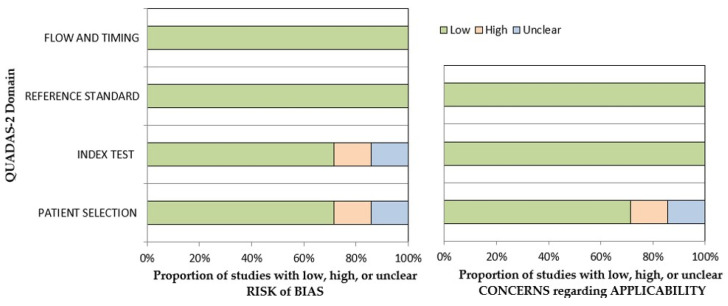
Concerns graph for all the seven studies included. QUADAS-2 shows the percentage of reports with low, high, and unclear risks of bias, and with low, medium, and high concerns for applicability, respectively.

QUADAS-2 revealed that the vast majority of the included research studies had a low risk of bias (Table S2). The risk of bias and applicability concerns graphs for all reports are depicted in Figure 2.

### 3.3. Diagnostic Accuracy of miR-375 in PCa

**Figure 3:**
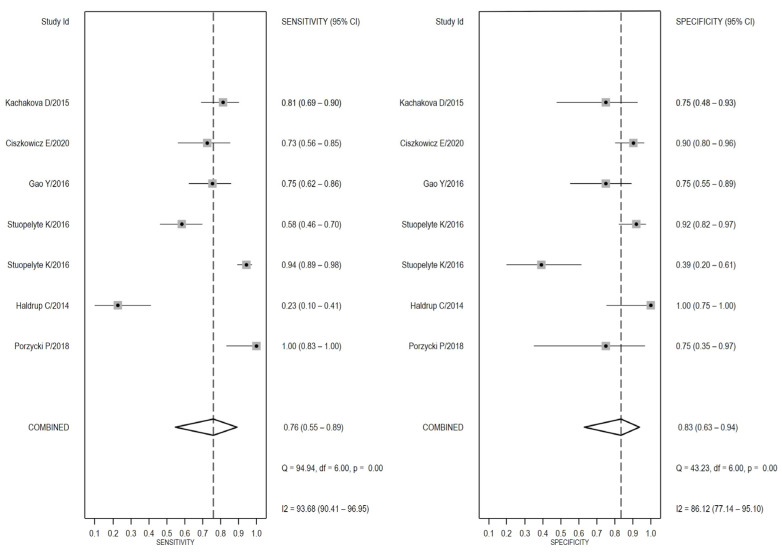
Sensitivity- and specificity-based forest plots in differentiating PCa patients from healthy/benign subjects. These plots display diagnostic probabilities with 95% CIs. Pooled estimates for specificity and sensitivity values are depicted as rhombus symbols [17,22,23,24,26,27].

**Figure 4:**
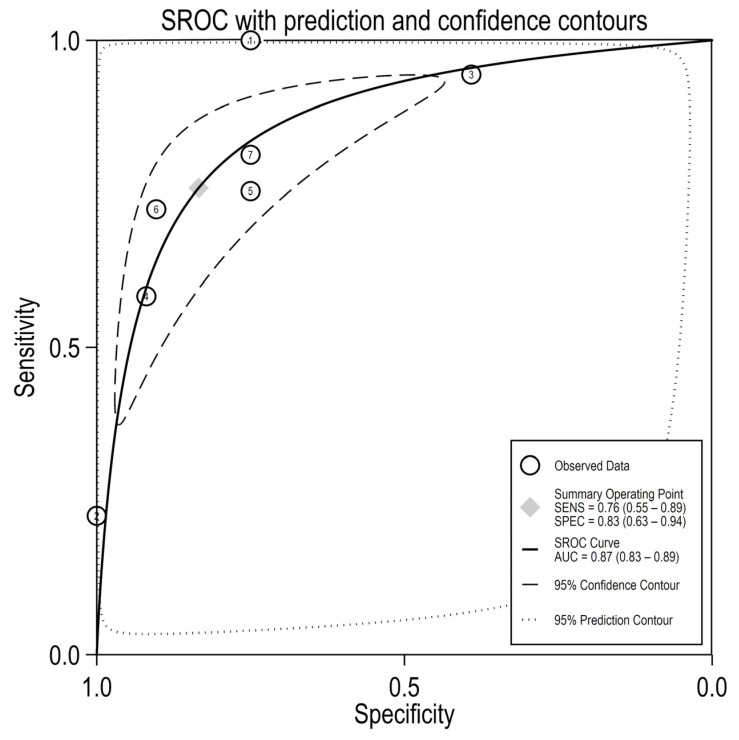
SROC curve for miR-375 in differentiating PCa from healthy/benign (with 95% CI and 95% prediction region). Circles represent estimates for each individual report (1 = Porzycki, 2 = Haldrup, 3 = Stuopelyte (1), 4 = Stuopelyte (2), 5 = Gao, 6 = Ciszkowicz, 7 = Kachakova).

Since, after testing for inter-study heterogeneity, we found a significant heterogeneity (I^2^ = 93.68% and 86.12% for sensitivity and specificity, respectively, Figure 3), we decided to use the random-effects model. Our meta-analysis showed a pooled sensitivity of 0.76 (95% CI: 0.55–0.89) and a pooled specificity of 0.83 (95% CI: 0.63–0.94) and an AUC value of 0.87 (95% CI: 0.83–0.89, Figure 4), which signifies an overall good diagnostic accuracy for miR-375.

Based on the calculated pooled estimates of sensitivity and specificity, we calculated the average likelihood ratios of the positive and negative test results and found that the LR+ and LR− of miR-375 were 4.6 (95% CI: 2.30–9.30) and 0.29 (95% CI: 0.16–0.51), respectively. In addition, we obtained an average DOR value of 16 (95% CI: 10–26).

Next, we investigated the possible heterogeneity sources in both sensitivity and specificity by performing a specimen-based meta-regression analysis, and we discovered that the types of biological specimen used might represent a potential heterogeneity source in terms of specificity (*p* < 0.001). However, given the small-scale design of our paper, additional reports are warranted to fully elucidate the source of the heterogeneity.

### 3.4. Sensitivity Analysis and Publication Bias

**Figure 5:**
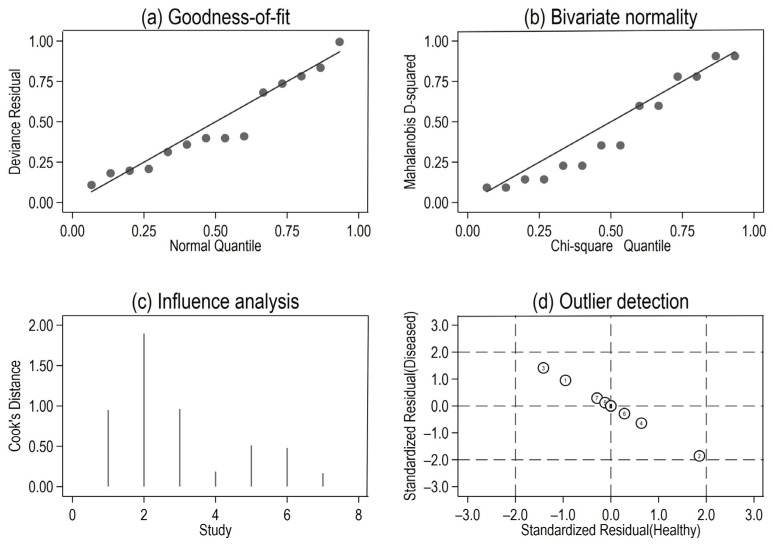
Capabilities for model checking: (**a**) goodness-of-fit based on residuals, (**b**) bivariate normality, (**c**) influence analysis, and (**d**) detection of outliers (1 = Porzycki, 2 = Haldrup, 3 = Stuopelyte (1), 4 = Stuopelyte (2), 5 = Gao, 6 = Ciszkowicz, 7 = Kachakova).

Both goodness-of-fit and bivariate normality analysis (Figure 5a,b) indicated the robustness of the calculation of the pooled estimates by the random-effects bivariate model, and no outliers were identified by influence analysis or in the outlier detection plot. (Figure 5c,d).

**Figure 6:**
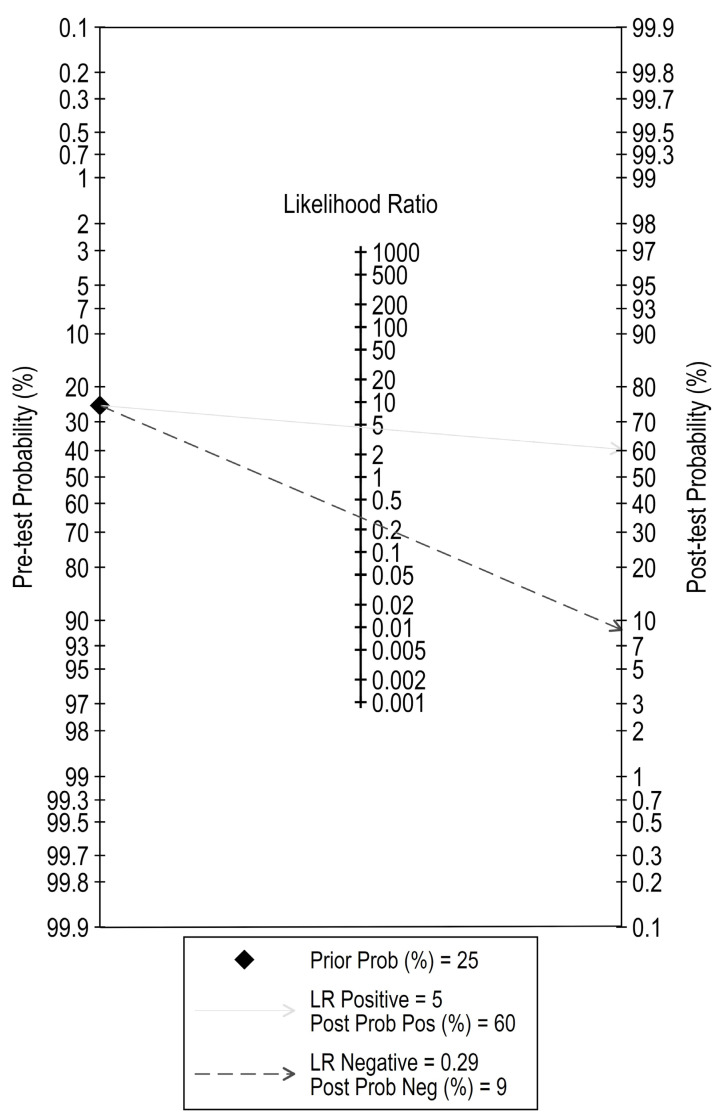
The evaluation of post-test probabilities using Fagan’s nomogram.

Analysis of pretest and posttest probabilities suggests a somewhat high value of miR-375 as a future promising diagnostic biomarker for this urologic disease. At a pretest probability of 25%, the posttest probability positivity would increase to 60% with a LR+ value of 5, whereas the posttest probability negativity would decrease to 9% with a LR− of 0.29, as seen in Figure 6 as well.

**Figure 7:**
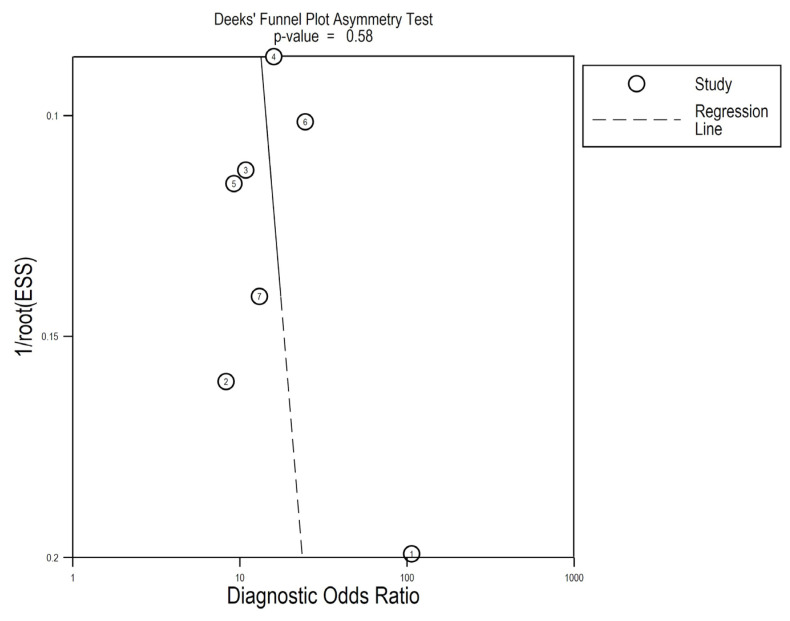
Deeks’ funnel plot for asymmetry linear regression test (1 = Porzycki, 2 = Haldrup, 3 = Stuopelyte (1), 4 = Stuopelyte (2), 5 = Gao, 6 = Ciszkowicz, 7 = Kachakova).

Lastly, we performed the pooled sensitivity and specificity analyses after the individual removal of each report, and we discovered that the final results did not differ to a great extent relative to the initial results. Correlated with the lack of significance in the Deeks’ asymmetry test (*p* = 0.58, Figure 7), these data highly suggest a lack of significant publication bias across reports and underline the stability and credibility of the results.

## 4. Discussion

In this study, we aimed to predict the true diagnostic value of miR-375 in PCa detection by combining seven studies (from six different articles, with one duplicate) that examined differences in circulating miR-375 expression levels of PCa patients’ samples (such as blood-derived samples and urine) relative to healthy volunteers BPH patients. The pooled sensitivity, specificity, and AUC values (0.76, 0.83, and 0.87, respectively) demonstrate that miR-375 possesses a high diagnostic accuracy in detecting PCa. The LR+ value of 4.6 indicates a somewhat satisfying ability to discriminate between cases and healthy, while 0.29 for the LR− value indicates a powerful capacity to eliminate healthy controls. The DOR value (16) reveals a surprisingly high diagnostic accuracy for miR-375 as well.

PCa is one of the most frequently over-diagnosed cancers in men (mainly due to the lack of specificity of currently used diagnostic biomarkers) and has therefore attracted particular interest among clinicians and researchers [35]. Novel biomarkers such as miRs are constantly being researched in terms of diagnostic values [36,37,38]. In particular, MiR-375 is well-documented in the literature in relationship with various diseases (PCa included), and it appears to be an oncogenic miRNA, as an increased miR-375 gene expression is positively correlated with high biochemical recurrence risk. In addition, Brase et al. demonstrated a correlation between miR-375 and other clinicopathological endpoints of PCa, describing an upregulation of miR-375 in PCa patients with advanced-stage disease, suggesting that miR-375 might distinguish metastatic PCa from healthy controls [39]. Hence, miR-375 has been repeatedly associated with metastatic PCa and general advanced-state disease; interestingly, the vast majority (72.9%) of the PCa cases included in our report were patients with early-stage disease (clinical/pathological stages 1 and 2). Therefore, by assessing the diagnostic value of miR-375 in this population, we have demonstrated that miR-375 could represent a potential diagnostic biomarker for early tumorigenesis phases as well. However, this remains an aspect that could benefit from future additional research encompassing larger cohort sizes and powerful statistical methods for the analysis of the high-throughput data.

To our knowledge, this is the first meta-analysis to evaluate the accuracy of miR-375 in PCa diagnosis alone. However, Yan et al. (2017) assessed the potential role of miR-375 in a broader meta-analysis encompassing multiple human cancers together with PCa. Although the aforementioned report encompassed more subjects, the true diagnostic utility of miR-375 remains disputable since the inclusion of various cancers within the same study design introduces considerable heterogeneity for the results to be accurately interpreted [40]. Other studies that focused on PCa and castration-resistant PCa analyzed two or more miRs (miR-375 included) in the same meta-analysis; the inclusion of a miR panel in a review report of this kind definitely provides finer and greater statistical power but further complicates the design of the biomarker tool and substantially increases the costs for the eventual laboratory tests [41,42].

Therefore, when narrowing the research inclusion criteria, heterogeneity arising from diverse types of cancer and miscellaneous sample population is considerably reduced although a major limitation remains the relatively decreased cohort size included for the downstream statistical analysis. Nonetheless, reported sensitivity and specificity values varied considerably across some studies in this meta-analysis design as well. Namely, the sensitivity values between the study conducted by Porzycky et al. and by Haldrup et al. were found to be highly discordant: 1 and 0.23 for the latter [17,22]. Interestingly, the Haldrup study had no biases following QUADAS-2 evaluation; therefore, the decreased sensitivity value could arise from the heterogeneity across the PCa population since the authors included in the comparison multiple PCa forms (localized, with local and/or distant metastases, and castration-resistant PCa vs. BPH). In addition, different extraction procedures were carried out between the two studies, which could have affected the yield and purity of total RNA retrieved for downstream PCR quantification. Furthermore, the Haldrup study was merely an expression-profiling study assaying multiple miRs from a human panel, while the Porzycky study was a validation study using specific forward and reverse primers for miR-375 expression individually. The choice of statistical analysis softwares might have differed between the two studies as well, hence the discordant sensitivity values, although it was not clearly reported by Haldrup et al.

In addition, miR-375 appears to represent an ideal biomarker due to its wide distribution in circulating samples (plasma, serum, and urine) and could therefore be screened in a minimally invasive fashion via liquid biopsy. This novel screening technique for blood-based biomarkers is of great interest for miR research, as high amounts of tissue-specific miRs can be easily found and collected from numerous biological fluids, decreasing the need for unnecessary biopsies and being able to detect minimal changes in the expression level of molecules such as miRs in early, asymptomatic stages of cancer, including PCa [43].

However, the exact mechanism of miR-375 in PCa is yet to be fully understood. Interestingly, it appears that miR-375 may play a dual role in PCa carcinogenesis, as in cells with low miR-375 levels (PC-3 cell lines), expression-induced miR-375 displayed reduced invasion, cell viability, and substantially increased apoptosis, while in high miR-375-expressing cells (22Rv1), the malignant phenotype was diminished by knockdown experiments [44]. Similar findings were confirmed by a separate, independent study analyzing miR-375-induced docetaxel resistance, where this miR proved to play dual roles as well. Increased miR-375 levels appeared to decrease sensitivity to docetaxel in vitro, while overexpression of miR-375 lead to apoptosis and cell growth inhibition. In vivo experiments performed on mouse xenograft models revealed that the cells with the highest docetaxel tolerance were the ones with increased miR-375 expression [45].

## 5. Conclusions

Given that PCa is a highly complex and heterogeneous disease warranting novel and more specific diagnostic biomarkers for early disease detection, we have demonstrated herein that miR-375 could represent a promising future biomarker for the screening diagnosis pathway of this urologic malignancy. Taken together, although miR-375 was highly studied in relationship with PCa pathophysiology, more in-depth studies are warranted to definitely assess its diagnostic biomarker value in PCa alone.

## Figures and Tables

**Table 1:** General characteristics of the seven studies included in our meta-analysis. Abbreviations: HC, healthy controls; BPH, benign prostatic hyperplasia; qPCR, quantitative polymerase chain reaction; sens, sensitivity; spec, specificity; TP, true positive; FP, false positive; FN, false negative; TN, true negative; AUC, area under the curve.

First Author	Porzycki [17]	Haldrup [22]	Stuopelyte [23]	Stuopelyte [23]	Gao [24]	Kachakova [26]	Ciszkowicz [27]
Year	2018	2014	2016	2016	2016	2015	2020
Specimen	serum	serum	urine	urine	plasma	plasma	serum
Cases	20	31	143	72	57	59	40
Controls	8 HC	13 BPH	23 BPH	62 HC	28 BPH	16 BPH	62 BPH
Method	qPCR	qPCR	qPCR	qPCR	qPCR	qPCR	qPCR
Endogenous Ctrl.	U6 SNORD44	UniSp3	Cel-miR-39	Cel-miR-39	U6	RNU6	RNU6
Dysregulation	up	up	up	up	up	down	up
Sens	1.00	0.23	0.94	0.58	0.75	0.81	0.73
Spec	0.75	1.00	0.39	0.92	0.75	0.73	0.90
TP	20	7	135	42	43	48	29
FP	2	0	14	5	7	4	6
FN	0	24	8	30	14	11	11
TN	6	13	9	57	21	12	56
AUC	0.906	0.65	0.6841	0.7968	0.757	0.809	0.892
AUC 95% CI	0.797–1.001	0.477–0.823	NA	NA	0.640−0.874	0.697−0.922	0.833−0.952

## Data Availability

Not applicable.

## Supplementary Materials

The following supporting information can be downloaded at: https://www.mdpi.com/article/10.3390/medicina58040529/s1, Table S1: PRISMA statement guidelines; Table S2: Quality assessment for Diagnostic Accuracy Studies-2 (QUADAS-2) for the included studies.
